# Antibacterial apple cider vinegar eradicates methicillin resistant *Staphylococcus aureus* and resistant *Escherichia coli*

**DOI:** 10.1038/s41598-020-78407-x

**Published:** 2021-01-20

**Authors:** Darshna Yagnik, Malcolm Ward, Ajit J. Shah

**Affiliations:** Department of Natural Sciences, School of Science and Technology, Middlesex University, The Burroughs, London, NW4 4BT England, UK

**Keywords:** Immunology, Microbiology, Health care

## Abstract

Methicillin-resistant *Staphylococcus aureus* (MRSA) and resistant *Escherichia coli* (r*E.coli*) infections can spread rapidly. Further they are associated with high morbidity and mortality from treatment failure. Therapy involves multiple rounds of ineffective antibiotics alongside unwanted side effects, alternative treatments are crucial. Apple cider vinegar (ACV) is a natural, vegan product that has been shown to have powerful antimicrobial activity hence we investigated whether ACV could ameliorate these resistant bacteria. The minimum dilution of ACV required for growth inhibition was comparable for both bacteria (1/25 dilution of ACV liquid and ACV tablets at 200 µg/ml were effective against r*E. coli* and MRSA). Monocyte co-culture with microbes alongside ACV resulted in an increase in monocyte phagocytosis by 21.2% and 33.5% compared to non-ACV treated but MRSA or *rE. coli* stimulated monocytes, respectively. Label free quantitative proteomic studies of microbial protein extracts demonstrated that ACV penetrated microbial cell membranes and organelles, altering the expression of key proteins. This resulted in significant reductions in total protein expression, moreover we could only detect ribosomal proteins; 50 s 30 s, enolase, phosphenol pyruvate and the ATP synthase subunit in *rE. coli*. Elongation factor iNOS and phosphoglycerate kinase OS were the only proteins present in MRSA samples following ACV treatment.

## Introduction

Microbial infections are presently posing major health threats worldwide on inconceivable scales. In fact, the globe is amid a deadly (coronavirus) COVID-19 pandemic and there is no cure. Infections such as HIV, SARS-CoV2, E-bola, MRSA and *E. coli* have been shown to spread rapidly among humans, causing complications including sepsis accompanied by cytokine storms which can lead to multiple organ failure and fatalities^1^. In addition, antimicrobial resistance and viral mutations are a huge concern which has led to further limits on therapeutic choice The World Health Organisation’s (WHO) 2017 global report on antimicrobial resistance states that there are high resistance rates to last generation drugs commonly used to treat serious hospital infections. It also highlights a two fold increase in all-cause mortality for both cephalosporin resistant and fluoroquinolone-resistant *E. coli*^2^.

The inappropriate use of antibiotics by humans, animal farms, poor hygiene and the lack of control of infections in healthcare have contributed to the emergence of antibiotic resistant bacteria. Multidrug resistant and extended spectrum beta lactamases producing *E. coli* can cause life threatening infections. *E. coli* and *Staphylococcus aureus* forms part of the facultative flora in the gastrointestinal tract and skin respectively^3^. Antibiotic resistance develops after prolonged exposure to antibiotics. Symptoms of *E. coli* infections include bloody diarrhoea, nausea, vomiting, dehydration, abdominal cramping and can lead to septic shock or death^4^. MRSA infection continues to be a major public health concern worldwide especially in hospitals and healthcare settings. MRSA can be fatal in immunocompromised, cancer patients, the elderly and even during routine hospital surgery. It spreads rapidly causing significant complications. MRSA can produce numerous toxins including super antigens that can lead to toxic shock syndrome and staphylococcal scarlet fever. Symptoms of MRSA infection often includes multiple swollen skin boils, rashes, headaches, chills, fever, fatigue, cough, breathlessness, and chest pain. Complications can lead to gangrene, painful abscesses and infections of the blood, cardiac, lung or urinary tract systems^5–7^. Although methicillin is no longer prescribed, MRSA tends to have antibiotic resistance to penicillin, cephalosporins, carbapenems and can develop resistance to quinolones, aminoglycosides and macroglides^8^. It was in the UK and Denmark in the 1960’s that the first MRSA epidemic occurred leading to the first MRSA isolates occurred^9^.

ACV is made from an alcoholic fermentation process of a combination of apples, sugar and yeast. The constituents include 5% acetic acid, mother of vinegar enzymes as well as potassium, magnesium and calcium^10,11^. One of these ingredients acetic acid, has potent antimicrobial properties and has been shown to inhibit planktonic growth of biofilms consisting of *A. baumannii* and *P. aeruginosa* on burns^12^. It has also been shown to inhibit yeast cell growth by causing mitochondrial and ribosomal degradation leading to apoptosis^13^. We have previously shown that ACV has strong antimicrobial action against non-resistant *E. coli*, *Candida albicans* and *Staphylococcus aureus*^14^. Therefore, the aim of the present study was to investigate the antibacterial activity of ACV on antibiotic resistant microbes.

## Results

### Antimicrobial activity of ACV against resistant *E.coli and MRSA*

To determine the anti-microbial activity of ACV we directly cultured resistant *E. coli* or MRSA with different concentrations of ACV.

**Figure 1:**
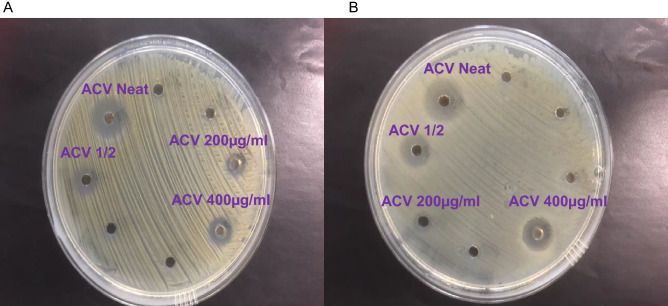
Effect of ACV on microbial growth after incubation at 37° C for 24 h on agar plates. (**A**) Resistant *E. coli*; (**B**) MRSA. ACV was either applied neat, 1:2 v/v in distilled water or ACV tablets at 400 or 200 μg/ml. Photographs were taken using a 20 Mega pixel Samsung camera.

**Figure 2:**
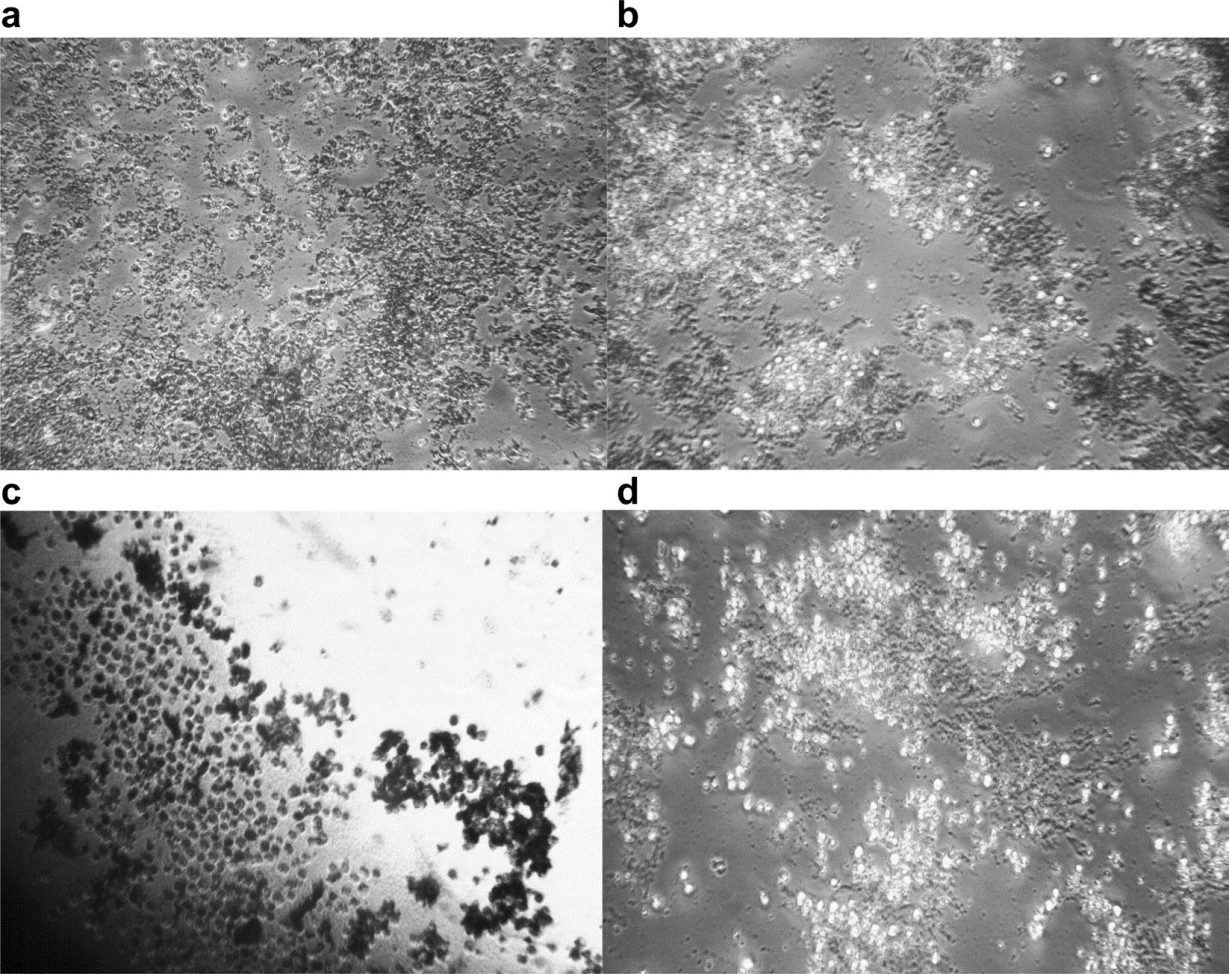
Light microscopic images representing the effect of ACV on co-cultures of monocytes with microbes after incubation for 24 h at 37 °C Cell viability was assessed using trypan blue staining. **(A)** MRSA, **(B)** MRSA with ACV, **(C)** resistant *E. coli*; **(D)** resistant *E. coli* with ACV.

Figure 1 shows the inhibition of growth in MRSA and *rE. coli* after treatment with different concentrations of ACV from (neat or undiluted, 1:2, 1:10, 1:25 v/v of Bragg’s liquid ACV or ACV tablets dissolved in water at 200 and 400 µg were used in culture with the microbes). Figure 2 represents light microscopic images of the co-culture of monocytes with both microbes with and without ACV (1:25 dilution) stained with trypan blue. To note there were significantly more monocytes which were alive following ACV addition as well as exposure to microbes. Furthermore, even neat ACV addition to monocytes in culture for 6 h was not toxic to the cells with over 90% monocyte viability. The minimum dose required to restrict growth for MRSA and *rE. coli* was a 1:25 dilution of Bragg’s ACV (0.5% acetic acid). We also compared different doses of commercial ACV oral tablets on the microbes. We tested concentrations in the range 50-400ug/mL in doubling dilutions of the ACV tablets against each microbe. The MIC for ACV tablets was 200 µg/mL for both MRSA and *rE. coli* respectively (Table 1). All dilutions were carried out using sterile water. We used Braggs ACV for all future experiments at the minimum inhibitory concentration required for each organism.

**Table 1:** Microbe growth inhibition zone measurements after exposure to ACV and antibiotics.

Microbe	Zone of inhibition (mm) (mean ± SD)
Resistant *E. coli* + ACV (neat)	8 ± 1
Resistant *E. coli* + ACV (1 in 2)	4.67 ± 0.5
Resistant *E. coli* + ACV (1 in 10)	1.57 ± 0.5
Resistant *E. coli* + ACV (1 in 25)	0.33 ± 0.6
Resistant *E. coli* + ACV (1 in 50)	0
MRSA + ACV (neat)	8.4 ± 0.7
MRSA + ACV (1 in 2)	4.38 ± 0.5
MRSA + ACV (1 in 10)	1.5 ± 0.6
MRSA + ACV (1 in 25)	0.25 ± 0.5
MRSA + ACV (1 in 50)	0
Resistant *E. coli* + ACV (400 µg/mL)	5.6 ± 1
Resistant *E. coli* + ACV (200 µg/mL)	3.33 ± 0.6
Resistant *E. coli* + ACV (100 µg/mL)	0.33 ± 0.6
Resistant *E. coli* + ACV (50 µg/mL)	0
MRSA + ACV (400 µg/mL)	7.8 ± 0.6
MRSA + ACV (200 µg/mL)	3.5 ± 0.6
MRSA + ACV (100 µg/mL)	1 ± 0.8
MRSA + ACV (50 µg/mL)	0
MRSA + clindamycin (125 mg/mL)	10.18 ± 0.8
Resistant *E. coli* + trimethoprim (150 mg/mL)	9.8 ± 0.5

*E.coli* or MRSA were inoculated onto MHA plates and co-cultured with different concentrations of ACV or antibiotics at 37° C for 24 h. After which time the effect on microbial growth was measured in mm by the diameter of the inhibition produced. Data is presented as mean ± SD of three similar experiments.

### Upregulation of phagocytic capacity

Monocytes ingest microbes primarily through phagocytosis hence we tested the effects of ACV on this process.

Indeed a 21.2%, and 33.5% increase in monocyte phagocytic capacity was observed after MRSA or *rE. coli* co-culture with ACV (1:25 concentration) respectively and in comparison, to the resting unstimulated monocytes. The results were comparable to commonly prescribed antibiotics to these infections such as clindamycin or trimethoprim. Results are expressed as the mean and SD of three similar experiments (Table 2).

**Table 2:** Effect of ACV on human monocyte phagocytic capacity.

Monocytes co-cultured with microbes	% Phagocytosis (mean ± SD)
Resistant *E. coli*	19.6 ± 0.8
Resistant *E. coli* + ACV (1/25)	29.5 ± 1.0
MRSA	26.9 ± 1.9
MRSA + ACV (1/25)	33.0 ± 2.4
MRSA + clindamycin (125 mg/mL)	34.5 ± 0.5
Resistant *E. coli* + trimethoprim (150 mg/mL)	32.6 ± 0.7

In vitro differentiated macrophages were incubated with microbes for 4 h at 37 °C. Cells cultured with microbes with or without ACV were washed and processed for detection on the Beckton Dickinson flow cytometer. An analysis of changes in regional gated profiles and % shift in side scatter was measured. Data is presented as mean ± SD of 3 similar experiments.

Proteomic results of *rE. coli* and MRSA after exposure to ACV. Label free quantitative bottom-up proteomic study of microbes cultured with and without ACV were directly compared. We were able to identify more than 200 proteins of both microbe proteomes (supplement Table 1). In ACV treated *rE. coli* cultures there was an absence of key enzymes together with DNA replication, glycolytic and respiratory proteins. We could only detect a handful of proteins such as ATP synthase subunit, enolase, phosphenol pyruvate and ribosomal proteins 30 s, 50 s (Table 3). MRSA cultures were incredibly sensitive to ACV treatment. Key enzymes which were missing after ACV treatment included phosphoglycerate and chaperone protein elongation factor proteins compared to the total 200 MRSA proteins identified in controls (Table 3).

**Table 3:** List of *E. coli* proteins identified following ACV treatment. *E. coli* were cultured with 1/25 dilution of ACV or alone in broth for 24 h at 37 °C in a shaking incubator.

Microbe	Proteins absent after ACV treatment from microbe proteome analysis
Resistant *E. coli* + ACV (1/25)	Ribosomal proteins 30 s, DNA directed RNA polymerase subunit alpha, Elongation factor TU, Elongation factor G OS-*E. coli*, formate acetyl transferase 1 OS E-coli, chaperone protein, 60 kDa chaperonin OS *E. coli*
MRSA + ACV (1/25)	Elongation factor TU, phosphoglycerate kinase

Proteins were extracted, digested and subsequently analysed using ultra high performance liquid chromatography with high resolution mass spectrometry. The proteome for each organism was detected and the absence of proteins following 24 h of ACV treatment were identified using Proteome Discoverer software and tabulated. Data represent the repeats of 12 such experiments.

## Discussion

Millions of years of evolution have resulted in bacteria evolving their survival utilising complex mechanisms of drug resistance to avoid killing by antimicrobial agents. ACV has grown in popularity forming the basis of a multitude of commercial health drinks and has been implicated as a cure all for many maladies^15^.

Our previous in vitro study highlighted the anti-inflammatory as well as antimicrobial effect of ACV on non-resistant *E. coli, S. aureus and C. albicans*^14^. We now extend the antimicrobial activity of ACV against MRSA and *rE. coli*. ACV inhibition of bacterial growth is comparable to the inhibition shown by trimethoprim and clindamycin which are typical antibiotics used to treat clinical *E. coli* and MRSA infections respectively^16,17^. Proteomic analysis of ACV treated resistant *E-coli* revealed the presence of key ribosomal proteins (30 s, 50 s). Interestingly erythromycin and other macrolide antibiotics can also target ribosomal structures which are involved in elongation structural polypeptides^18^. Perhaps this indicates the robustness of the ribosomal *E. coli* subunit. The presence of ATP synthase in *rE. coli* after ACV treatment is significant since ATP synthases are found ubiquitously in energy-transducing membranes of bacteria which are a crucial antibiotic target^19^. ACV could have destroyed the bacteria exposing its membrane where this protein is found. Bacterial enolase and phosphenol pyruvate were also detected following ACV treatment. Both proteins play a key role in bacterial growth and reproduction^20,21^. On ACV treated MRSA we could only identify two proteins against an extensive array of classified proteins of the MRSA genome. One of which was bacterial elongation factor which is a key translational protein involved in MRSA resistance and a focus of new antimicrobials^22^. The absence of phosphoglycerate kinase in MRSA following ACV addition indicates that ACV can disrupt the glycolytic MRSA pathway^23^. The anti-microbial capabilities of ACV have been verified clinically in a case of vaginal candida^24^. Recently ACV has been shown to have the capability of upregulating key innate immune defense genes in shrimp as well as increasing their growth in vivo following consumption. It has also been shown to support the intestinal shrimp microbiome^25,26^. A study in which diabetics were given ACV orally found that it increased the activity of antioxidant enzymes such as catalase and dismutase and glutathione peroxidase^27^. This could be an important factor in its antimicrobial attributes. Antibiotic resistant bacteria can cause complications especially in the gut^28^. In fact, it has been shown that loss of colonization resistance can lead to an increase in vancomycin-resistant *E. coli* predisposing patients to blood sepsis^29^. Hence, any compound which can amplify the innate immune system could be a formidable antidote for these dreaded resistant organisms. Another component of ACV which could account for its antimicrobial action is acetic acid which has been shown to be active at high (1.8%) and low concentrations including 0.3%^12^. In our studies the minimum ideal concentration for activity was a 1:50 dilution which would contain 0.1% acetic acid. Hence acetic acid may not be the only antimicrobial active component in ACV. Low pH can also effectively destroy microbes. ACV is mildly acidic with a pH of 5.6 compared to acetic acid which has a pH of 4.7. We did investigate the effects of strong organic acids such as hydrochloric and sulphuric acid at a range of concentrations on different microbes (results not shown) but did not see the same antimicrobial effects as with ACV. The effect of ACV on human blood parameters is scarce. We demonstrate for the first time that ACV did not have any effects on human monocytes in cultures, even after 24 h of direct ACV exposure at high concentrations; verifying that it is not damaging to white blood cells. This is a vital observation since ACV has been consumed as a food supplement for centuries, many people take it daily hence any side effects are important to highlight. ACV had toxic effects on microbes but reassuringly did not affect the integrity of human monocytes in our experiments. To note ACV should be consumed diluted in a little water or juice as it could burn the throat, otherwise no known toxicity or side effects have been reported due to its consumption. A study by Oussaid et al. 2020 investigated the effects of simultaneous glucose and ACV on rats who were fed 2 mL of ACV per kg of their weight for five weeks. There was an increase in plasma urea levels in the rats that were fed glucose alone but no change in urea levels in ACV treated rats. This demonstrated ACV did not affect kidney function in fact it corrected the urea levels when fed in combination with glucose^29^.

We conclude that ACV can have powerful anti-microbial effects directly on resistant *E. coli* and MRSA. Mode of action seems to involve an alteration in the pathogenic physiology of the microbes. These in vitro results highlight the antimicrobial capacity of ACV. Therefore, this study prompts further clinical research into the efficacy of treating patients infected with resistant bacterial infections with ACV supplementation. ACV could form the core ingredient of a contemporary pharmacological antimicrobial against MRSA and *rE. coli*.

## Materials and methods

### Chemical reagents, microorganisms, media and culture conditions

Microbial strains: resistant *E. coli* strain (NCTC 13353) which is highly resistant to cefepime and cefepime-enmetazobactam combination was purchased from LGC Promochem. MRSA strains (ATCC 33591, NCTC8325 resistant to methicillin) were purchased from Public Heath, Colindale, England. U937 cell line CRL-1593.2 was purchased from the American type culture collection.

### Reagents

Dulbecco’s modified iscoves media, dimethyl-ethyl-sulphonyl-oxide, HANKS balanced salt solution, histopaque, ethanol, phosphate buffered saline, paraformaldehyde, acetone, dithiothreitol, iodoacetamide, trypsin from porcine pancreas of proteomics grade, formic acid, acetonitrile, HPLC-grade water, methanol and Whatman Mini-UniPrep syringeless filter devices (pore size 0.45 µm) were purchased from Sigma Aldrich (Poole, U.K.). TNF-alpha, enzyme linked immunosorbent assays (ELISA’s) were purchased from Research and Development Systems (Abingdon, U.K.). Mueller Hinton agar was purchased from Oxoid, UK. Bragg’s Apple Cider Vinegar and apple cider vinegar tablets (500 mg) were purchased from Troo healthcare, Colchester, UK.

### Inoculum preparation and measurement of anti-microbial activity of ACV which was based on previously published methods by Yagnik et al.^14^

Cultures of resistant *E. coli* and MRSA were grown in nutrient media. All cultures were cultivated in a shaking incubator at 37 °C for 24 h overnight prior to use. Mueller Hinton agar (MHA) was prepared by dissolving 38 g in 1 L of distilled water, boiling the mixture for 1 min, after cooling and autoclaving, it was poured into plates. These were left to dry and subsequently stored at 37 °C. All microbial cultures were adjusted to 0.5 McFarland’s standard 1.5 × 10^8^ CFU/ml and 4 × 10^6^ CFU/ml of each organism were used in experiments. Each microbe was swabbed evenly onto plates containing MHA. For sample addition, 100 µL of ACV at varying concentrations was added to the wells which were punched into the agar. The plates were then incubated at 37 °C for 24 h. Zones of inhibition surrounding samples were identified, photographed and measured in mm. Experiments were repeated at least five times.

### Ethical approval

All experimental protocols were approved by the Middlesex University Natural Sciences Ethics Committee, UK number 2323. In addition, the methods were carried out in accordance with the relevant guidelines and regulations.

### Human mononuclear cell culture as similarly published previously by Yagnik et al.^14^

The human monocyte cell line U937 was used as a source of monocytes purchased from American type culture collection (ATCC). Cells were cultured at a density of 4 × 10^5^/ml with either *rE. coli* , MRSA or varying concentrations of ACV at counts of 4 × 10^6^ CFU/ml respectively for 24 h at 37 °C and 5% CO_2_ after which supernatants were collected and analysed for TNF-α secretion using ELISA kits following manufacturer’s protocols.

### Monocyte phagocytic capacity measurement by flow cytometry

U937cells were cultured at 4 × 10^5^/ml in 24 well plates over a period of two days after which they were incubated with microbes (4 × 10^6^ CFU/ml) for 4 h at 37 °C and 5% CO_2_. Cells were then scraped replenished in ice cold PBS containing 1 mM EDTA, washed and removed from plates. The resultant pellets were fixed in 400 µL of 4% paraformaldehyde and analysed using a FACS Calibur flow cytometer (Beckton Dickinson Immunocytometry Systems, UK and *Cell Quest* software 2019).

### Sample preparation for mass spectrometry analysis as similarly published previously by Yagnik et al.^14^

MRSA and *rE. coli* was incubated with or without ACV at 1/25 dilution for 24 h at 37 ∘C and centrifuged at 900 rpm, supernatant was removed leaving bacteria pellets. At least 12 sample repeats were used. Bacteria cell pellets were resuspended in cold acetone (600 μL) and centrifuged at 14,800 rpm for 5 min to precipitate protein. The supernatant was removed and 50 mM ammonium bicarbonate (400 μL) was then added to dissolve the extracted proteins. The protein concentration was measured using a nano drop device (Thermo Fisher UK).

Samples were reduced with 10 mM dithiothreitol (DTT) for 15 min at 56 °C and alkylated with 100 mM iodoacetamide for 30 min at room temperature in the dark. Each sample was then digested with trypsin at 37 °C overnight.

### Mass spectrometry methods for microbial analysis based on those published previously by Yagnik et al.^14^

#### LC–MS/MS data acquisition based on methods similarly published previously by Yagnik et al.^14^

Tryptic digests were analysed using a Dionex Ultimate 3000 RSLC Nano ultra-high-performance liquid chromatography system coupled to a Q Exactive. Aliquot (15 μL) of tryptic digest was desalted and concentrated using a 5 mm × 300 μm i.d. C18 trap cartridge and solvents composed of a mixture of water and acetonitrile (98:2%, v/v) containing 0.05% TFA (loading solvent A) and a mixture of acetonitrile and water (80:20%, v/v) (loading solvent B) at a flow rate of 20 μL/min was used. The concentrated sample was separated using a binary gradient elution profile composed of a mixture of water and acetonitrile (95:5,v/v) containing 0.1% formic acid (eluent A) and a mixture of acetonitrile and water (80:20%, v/v) (eluent B) containing 0.1% formic acid at a flow rate of 6 μL/min. The gradient was 0 min—0% B, 4 min—5% B, 5 min—8% B, 40 min—40% B, 41 min—80% B. The auto sampler and column oven temperature were set to 4 and 40 °C respectively. The Q Exactive was operated in a data dependent mode. MS survey scans were acquired from *m/z* 350 to 2000 at resolution of 70,000 with AGC of 3e6 and maximum IT of 100 ms. The 10 most abundant ions were subjected to MS/MS and measured with a resolution of 17,500 and AGC of 1e5 and maximum IT of 200 ms^14^.

#### Data processing and analysis based on methods similarly published previously by Yagnik et al.^14^

LC–MS/MS data was processed using Proteome Discoverer v2.1 (ThermoFisher Scientific, UK) with database searching against a downloaded FASTA file originating from Uniprot_SwissProt_2019_02. Search parameters were set to match MS/MS spectra to peptides created by fully specific trypsin cleavage within a precursor mass tolerance of 10 ppm and with a fragment mass tolerance of 0.02 Da. Carbamidomethyl (CAM: + 57.021 Da) was set as a static modification to cysteines whereas methionine oxidation (MetOx: + 15.995 Da) was set as a variable modification. Results were initially visualised within the software and then exported to Excel for further review^14^.

### Statistical analysis

All experimental results are expressed as the mean ± standard deviation (SD). Statistical analyses were carried out using one way ANOVA or students t-test, outcomes were considered significant where p < 0.05 (when comparing apple cider vinegar treated microbes to the untreated groups in all experiments). All experiments were repeated at least 3–5 times. Analysis was carried out using Excel software version 2019.

## Supplementary Information

Supplementary Table 1.

## Data Availability

The datasets generated and analyzed during the current study can be made available upon request.

## References

[1] World Health Organization (WHO). Coronavirus disease (COVID-19). https://www.WHOint/emergencies/diseases/novel-coronavirus-2019. (2020).

[2] World Health Organization (WHO). *Global Priority List of Antibiotic-Resistant Bacteria to Guide Research, Discovery, and Development of New Antibiotics*. (WHO, Geneva, 2017).

[3] Tickell KD (2020). The effect of acute malnutrition on enteric pathogens, moderate-to-severe diarrhea, and associated mortality in the Global Enteric Multicenter Study cohort: A post-hoc analysis. Lancet Glob. Health..

[4] Kobayashi K (2018). Prediction of surgical site infection in spine surgery from tests of nasal MRSA colonization and drain tip culture. Eur. J. Orthop. Surg. Traumatol..

[5] Lee A (2018). Methicillin-resistant *Staphylococcus aureus*. Nat. Rev. Dis. Primers.

[6] Lowy FD (1998). *Staphylococcus aureus* infections. N. Eng. J. Med..

[7] Thakkar V, Ghobrial GM, Maulucci CM (2014). Nasal MRSA colonization: Impact on surgical site infection following spine surgery. Clin. Neurol. Neurosurg..

[8] Chambers HF (2009). Waves of resistance: *Staphylococcus aureus* in the antibiotic era. Nat. Rev. Microbiol..

[9] Jevons M (1961). “Celbenin”-resistant staphylococci. BMJ.

[10] Del Campo G (2008). A development of alcoholic and malolactic fermentations in highly acidic and phenolic musts. Bioresour. Technol..

[11] Medina, E. *et al*. Tackling threats and future problems of multidrug-resistant bacteria. *Curr. Top. Microbiol. Immunol.*10.1007/82_2016_492. (2016) 10.1007/82_2016_492 27406189

[12] Halstead (2015). The antibacterial activity of acetic acid against biofilm-producing pathogens of relevance to burns patients. PLoS ONE.

[13] Dong, Y., Hu, J., Fan, L., Chen, Q. RNA-Seq-based transcriptomic and metabolomic analysis reveal stress responses and programmed cell death induced by acetic acid in *Saccharomyces cerevisiae*. *Sci. Rep*. **17**(7), 42659, 10.1038/srep42659 (2017) 10.1038/srep42659 PMC5314350 28209995

[14] Yagnik, D., Serafin, V. & Shah, A. Antimicrobial activity of apple cider vinegar against *Escherichia coli*, *Staphylococcus aureus* and *Candida albicans*; downregulating cytokine and microbial protein expression. *Sci. Rep.*10.1038/s41598-017-18618-x (2018) 10.1038/s41598-017-18618-x PMC5788933 29379012

[15] Naziroglu M (2014). Apple cider vinegar modulates serum lipid profile, erythrocyte, kidney and liver membrane oxidative stress in ovariectomized mice fed high cholesterol. J. Membr. Biol..

[16] Duployez, C. *et al*. Trimethoprim susceptibility in *E. coli* community-acquired urinary tract infections in France. *Med. Mal. Infect.*10.1016/j.medmal.2018.03.010 (2018). 10.1016/j.medmal.2018.03.010 29673879

[17] Tong SY (2015). *Staphylococcus aureus* infections: Epidemiology, pathophysiology, clinical manifestations, and management. Clin. Microbiol. Rev..

[18] Chittum, H.S. *et al*. Erythromycin inhibits the assembly of the large ribosomal subunit in growing *Escherichia coli* cells. *Curr. Microbiol.* **30**, 273–9 (1995). 10.1007/BF00295501 7766155

[19] Nakanishi-Matsui M (2016). ATP synthase from *Escherichia coli*: Mechanism of rotational catalysis, and inhibition with the ε subunit and phytopolyphenols. Biochem. Biophys. Acta..

[20] Krucinska J (2019). Functional and structural basis of *E. coli* enolase inhibition by SF2312: A mimic of the carbanion intermediate. Sci. Rep..

[21] Arifin Y (2016). Escherichia coli W shows fast, highly oxidative sucrose metabolism and low acetate formation. Appl Microbiol Biotechnol..

[22] Leeds JA (2011). In vitro and in vivo activities of novel, semisynthetic thiopeptide inhibitors of bacterial elongation factor Tu. Antimicrob. Agents Chemother..

[23] Roychowdhury, A. *et al*. Expression, purification, crystallization and preliminary X-ray diffraction studies of phosphoglycerate kinase from methicillin-resistant *Staphylococcus aureus* MRSA252. *Acta Crystallogr. Sect. F Struct. Biol. Cryst. Commun*. 10.1107/S1744309111007391 (2011) 10.1107/S1744309111007391 PMC3107138 21636907

[24] Oxzen, B. *et al*. Vaginal candidiasis infection treated using apple cider vinegar: A case report. *Altern. Ther. Health Med*. (epub 2017 Nov 7) (2017). 29112940

[25] Pourmozaffar S, Hajimaradloo H, Mindare HK (2017). Dietary effect of apple cider vinegar and propionic acid on immune related transcriptional responses and growth performance in white shrimp, *Liopenaeus vannamei*. Fish Shellfish Immunol..

[26] Pourmozaffar S (2018). Effect of dietary supplementation with applecidervinegar and propionic acid on hemolymph chemistry, intestinal microbiota and histological structure of hepatopancreas in white shrimp, *Litopenaeus vannamei*. Fish Shellfish Immunol..

[27] Hmad HB (2019). Antidiabetic and antioxidant effects of apple cider vinegar on normal and streptozotocin-induced diabetic rats. Int. J. Vitam. Nutr. Res..

[28] Keith J (2020). Impact of antibiotic-resistant bacteria in the mouse intestine on immune activation and *Clostridioides difficile* infection. Infect Immun..

[29] Ousaaid D, Laaroussi H, Bakour M (2020). Beneficial effects of apple vinegar on hyperglycemia and hyperlipidemia in hypercaloric-fed rats. J. Diabetes Res..

